# The Human–Nature Relationship and Its Impact on Health: A Critical Review

**DOI:** 10.3389/fpubh.2016.00260

**Published:** 2016-11-18

**Authors:** Valentine Seymour

**Affiliations:** ^1^Department of Civil, Environmental and Geomatic Engineering, University College London, London, UK

**Keywords:** human health, human–nature relationship, natural environment, interdisciplinary

## Abstract

Within the past four decades, research has been increasingly drawn toward understanding whether there is a link between the changing human–nature relationship and its impact on people’s health. However, to examine whether there is a link requires research of its breadth and underlying mechanisms from an interdisciplinary approach. This article begins by reviewing the debates concerning the human–nature relationship, which are then critiqued and redefined from an interdisciplinary perspective. The concept and chronological history of “health” is then explored, based on the World Health Organization’s definition. Combining these concepts, the human–nature relationship and its impact on human’s health are then explored through a developing conceptual model. It is argued that using an interdisciplinary perspective can facilitate a deeper understanding of the complexities involved for attaining optimal health at the human–environmental interface.

## Introduction

During the last century, research has been increasingly drawn toward understanding the human–nature relationship (1, 2) and has revealed the many ways humans are linked with the natural environment (3). Some examples of these include humans’ preference for scenes dominated by natural elements (4), the sustainability of natural resources (5, 6), and the health benefits associated with engaging with nature (7–9).

Of these examples, the impacts of the human–nature relationship on people’s health have grown with interest as evidence for a connection accumulates in research literature (10). Such connection has underpinned a host of theoretical and empirical research in fields, which until now have largely remained as separate entities.

Since the late nineteenth century a number of descriptive models have attempted to encapsulate the dimensions of human and ecosystem health as well as their interrelationships. These include the Environment of Health (11), the Mandala of Health (12), the Wheel of Fundamental Human Needs (13), the Healthy Communities (14), the One Health (15), and the bioecological systems theory (16). Each, however, have not fully incorporated all relevant dimensions, balancing between the biological, social, and spatial perspectives (17, 18). In part this is due to the challenges of the already complex research base in relation to its concept, evidence base, measurement, and strategic framework. Further attention to the complexities of these aspects, interlinkages, processes, and relations is required for a deeper sense of understanding and causal directions to be identified (19).

This article reviews the interconnectivities between the human–nature relationship and human health. It begins by reviewing the each of their concepts and methodological approaches. These concepts will be converged to identify areas of overlap as well as existing research on the potential health impacts in relation to humanity’s degree of relationship to nature and lifestyle choices. From this, a developing conceptual model is proposed, to be inclusive of the human-centered perspective of health, viewing animals and the wider environment within the context of their relationship to humans. The model combines theoretical concepts and methodological approaches from those research fields examined in this review, to facilitate a deeper understanding of the intricacies involved for improving human health.

## Defining the Human–Nature Relationship

It is beyond the scope of this paper to review the various connections at the intersect of humanity and the natural environment. Instead, I summarize key concepts and approaches from those four research fields (Evolutionary Biology, Social Economics, Evolutionary Psychology, and Environmentalism) outlined below, which have paid most attention to studying this research area. I then summarize areas of convergence between these connections in an attempt to describe the human–nature relationship, which will serve as background to this review.

It is anticipated that through drawing on these different fields of knowledge, a deeper level of understanding can be brought to the growing issue of humanity’s relationship with nature and its impact on health. This is because examining the human–nature relationship from a single disciplinary perspective could lead to partial findings that neglect other important sources as well as the complexities that exist between interlinkages, causal directions, processes, and relations.

### Evolutionary Biology

Evolutionary biology is a branch of research that shortly followed Darwin’s (20) Theory of Evolution. It concerns the adaptive nature of variation in all animal and plant life, shaped by genetic architecture and developmental processes over time and space (21). Since its emergence over a century ago, the field has made some significant advances in scientific knowledge, but with intense debate still remaining among its central questions, including the rate of evolutionary change, the nature of its transitional processes (e.g., natural selection) (22). This in part owes to the research field’s interdisciplinary structure, formulated on the foundations of genetics, molecular biology, phylogeny, systematics, physiology, ecology, and population dynamics, integrating a diverging range of disciplines thus producing a host of challenging endeavors (23, 24). Spanning each of these, human evolution centers on humanity’s life history since the lineage split from our ancestral primates and our adaptive synergy with nature.

In the last four decades, evolutionary biology has focused much attention on the cultural–genetic interaction and how these two inherent systems interrelate in relation to lifestyle and dietary choices [*Culturgen Evolution* (25); *Semi-Independent* (26); *Dual-Inheritance model* (27)]. Some of the well-known examples include humans’ physiological adaptation to agricultural sustenance (28), the gradual increase in lactose tolerance (29) as well as the susceptibility of allergic diseases (e.g., asthma and hay fever) in relation to decreasing microbial exposure (30).

This coevolutionary perspective between human adaptation and nature has been further conceptualized by Gual and Norgaard (31) as embedding three integrated systems (biophysical, biotic, and cultural). In this, culture is both constrained and promoted by the human genetics *via* a dynamic two-way interaction. However, bridging the gap between these research fields continues to generate much controversy, particularly as the nature of these evolutionary development processes differs widely (e.g., internal and external factors). This ongoing discussion is fueled by various scholars from multiple disciplines. Some have argued that one cannot assume all evolutionary mechanisms can be carried over into other areas (32, 33), where genomes cannot evolve as quickly to meet modern lifestyle and dietary requirements (34). Conversely, others believe that humans have not entirely escaped the mechanisms of biological evolution in response to our cultural and technological progressions (35).

### Evolutionary Psychology

Evolutionary psychology is a recently developed field of study, which has grown exponentially with interest since the 1980s. It centers on the adaptation of psychological characteristics said to have evolved over time in response to social and ecological circumstances within humanity’s ancestral environments (36–38). This reverse engineering approach to understanding the design of the human mind was first kindled by evolutionary theorist Charles Darwin (20) in the last few pages of *Origin of Species*;

In the distant future … Psychology will be based on a new foundation, that of the necessary acquirement of each mental power and capacity by gradation [p. 447].

As such, evolutionary psychology is viewed by some to offer a metatheory that dissolves the traditional boundaries held in psychology (e.g., cognitive, social, personality, and development). Within this metatheory, all psychological theories implicitly believed by some to unify under this umbrella (37). However, the application of evolution to the study of psychology has not been without controversial debate in areas relating to cognitive adaptation, testability of hypotheses, and the uniformity of human nature (39).

During the past few decades, the field has presented numerous concepts and measures to describe human connectedness to nature. These include Deep Ecology (40), Extinction of Experience (41), Inclusion of Nature in Self (42), and Connectedness to Nature (43). However, the Biophilia hypothesis (44) remains the most substantially contributed to theory and argues for the instinctive esthetic preference for natural environments and subconscious affiliation for other living organisms. Supportive findings include humans’ preference for scenes dominated by natural elements (4), improved cognitive functioning through connectivity with nature (45) as well as instinctive responses to specific natural stimuli or cues (e.g., a common phobia of snakes) (46). More recently, evidence is emerging to suggest that connectivity to nature can generate positive impacts on one’s health, increasing with intensity and duration (47).

The underpinning of the Biophilia hypothesis centers on humanity’s source of attachment to nature beyond those on the surface particulars. Instead, it reflects thousands of years of evolutionary experience closely bonding with other living organisms (44). Such process is mediated by the rules of prepared and counter-prepared learning that shape our cognitive and emotional apparatus; evolving by natural selection *via* a cultural context (48). This innate value for nature is suggested to be reflected in the choices we make, experiences expressed as well as our longstanding actions to maintain our connection to nature (49). Nevertheless, many have gone on to recognize the research field’s need for revision and further evidentiary support through empirical analysis (50). Similarly, as other researchers have argued, these innate values should be viewed in complementary to other drivers and affinities from different sources that can also be acquired (e.g., technology and urban landscapes). This is because at the commonest level, as Orr (51) explains, humanity can learn to love what becomes familiar, a notion also reflected in the Topophilia (“love of place”) hypothesis (52).

### Social Economics

Social economics is a metadiscipline in which economics is embedded in social, political, and cultural behaviors. It examines institutions, choice behavior, rationality as well as values in relation to markets (53). Owing to its diverse structure, the human–nature relationship has been explored in various contexts. These include the reflections of society’s values and identities in natural landscapes (54), condition of placelessness (55), and humanity’s growing ecosynchronous tendencies (56) as well as how the relationship has evolved with historical context (57–59). While the dynamics of human and nature coupled systems has become a growing interdisciplinary field of research, past work within social economics has remained more theoretical than empirically based (59).

The connection between the start of industrialized societies and the dynamically evolving human–nature relationship has been discussed by many (60), revealing a host of economic–nature conflicts. One example includes those metaphorically outlined in the frequently cited article “*The Tragedy of the Commons*.” In this, it argues that the four laws of ecology are counter intuitive with the four laws of capitalism (5, 6). Based on this perspective, the human–nature relationship is simplified to one of exchange value, where adverse costs to the environment are rarely factored into the equation (6). However, this is not to say that humanity’s increasing specialization and complexity in most contemporary societies are distinct from nature but still depend on nature to exert (61).

Central to the tenets outlined in *Tragedy of the Commons* is the idea of “gradually diminishing freedom” where a population can increasingly exceed the limits of its resources if avoidance measures are not implemented (e.g., privatization or publicly owned property with rights of entry) (5, 62). Yet, such avoidance measures can be seen to reflect emerging arguments in the field of environmental justice, which researches the inequalities at the intersection between environmental quality, accessibility, and social hierarchies (63). These arguments derive from the growing evidence that suggests the human–nature relationship is seemingly disproportionate to those vulnerable groups in society (e.g., lack of green spaces and poor air quality), something public health researchers believe to be a contributing factor to health inequities (64). As such, conflicts between both private and collective interests remain a challenge for future social economic development (65). This was explored more fully in Ostrom’s (66) research on managing a common pool of resources.

### Environmentalism

Environmentalism can be broadly defined as an ideology or social movement. It focuses on fundamental environmental concerns as well as associated underlying social, political, and economic issues stemming from humanity’s interactions affecting the natural environment (67, 68). In this context, the human–nature relationship has been explored through various human-related activities, from natural resource extraction and environmental hazards to habitat management and restoration. Within each of these reflects a common aspect of “power” visible in much of the literature that centers on environmental history (69). Some examples included agricultural engineering (70), the extinction of animals through over hunting (71) as well as the ecological collapse on Easter Island from human overexploitation of natural resources, since disproven (72–74). Yet, in the last decade, the field’s presupposed dichotomy between humans and nature in relation to power has been critically challenged by Radkau (75) who regards this perspective as misleading without careful examination. Instead, they propose the relationship to be more closely in synchrony.

Power can be characterized as “*A person, institution, physical event or idea* … *because it has an impact on society: It affects what people do, think and how they live*” (76). Though frequently debated in other disciplines, in the context of the human–nature relationship, the concept of “power” can be exerted by both nature and humanity. In regards to nature’s power against humanity, it has the ability to sustain society as well as emphasize its conditional awareness, environmental constraints, and fragilities (77). In contrast, humanity’s power against nature can take the form of institutions, artifacts, practices, procedures, and techniques (70). In the context of this review, it focuses on nature’s powers against humanity.

It has been argued that human power over nature has altered and weakened in dominance (75) since the emergence of Rachel Carson’s book *Silent Spring* in 1962, and later concepts of Gaia (78), Deep Ecology (40), and Sustainable Development (79). Instead, humanity’s power toward nature has become one of a moral sense of protectionism or the safeguarding of the environment (80). This conservative behavior (e.g., natural defenses, habitat management, and ecological restoration) can be termed “Urgent Biophilia” (81) and is the conscious urge to express affinity for nature pending an environmental disaster. As Radkau (69) suggests, with warnings of climatic change, biodiversity loss, and depletions in natural resources, this poses a threat to humanity. As such, this will eventually generate a turning point where human power is overwhelmed by the power of nature, bringing nature and power into a sustainable balance. Nonetheless, as many also highlight, humanity’s responses to environmental disasters can directly impinge on an array of multi-causalities of intervening variables (e.g., resource depletion and social economics) and the complexity of outcomes (82).

### An Interdisciplinary Perspective of the Human–Nature Relationship

Through exploring the key concepts found in evolutionary biology, social economics, evolutionary psychology, and environmentalism, this has enabled a broader understanding of the various ways humans are connected to the natural environment. Each should not be viewed as separate entities, but rather that they share commonalities in terms of mutual or conjoint information and active research areas where similarities can occur (see Table 1 below). For example, there is a clear connection between social economics, evolutionary psychology, and biology in areas of health, lifestyle, and biophilic nature (40, 53, 81) as well as between social economics and the environment in regards to balancing relationships of power (5, 75). Similarly, economic–nature conflicts can occur between disciplines evolutionary psychology and social economics in relation to people’s affiliation for nature and industrial growth.

**Table 1:** **A summarized overview of human–nature relationship connections between those research fields explored**.

Research field	Type of connection	Description	Examples
Evolutionary biology	Cultural–genetic interaction (coevolution)	The interrelationship between two or more inherent systems (e.g., biophysical, biotic, and cultural). Examples used in this review related to lifestyle and dietary choices Overlaps identified between the following research disciplines and fields: human health (see Defining Health), genetics, evolutionary studies, culture, and social economic behaviors	Lumsden and Wilson (25); Boyd and Richerson (27); Cohen and Armelagos (28); Laland et al. (29); Bloomfield et al. (30); Gual and Norgaard (31); Simon (32); Nelson (33); Carrera-Bastos et al. (34); and Powell (35)
Evolutionary psychology	Affiliation to nature	The instinctive esthetic preference and value for nature. Examples used in this review related to people’s feelings of connectedness to nature Overlaps identified between the following research disciplines and fields: evolution, mental health and well-being (see Mental Health), social and behavioral ecology, psychology, culture, and human development	Wilson (44); Naess (40); Pyle (41); Schultz (42); Mayer and Frantz (43); Howell et al. (45); Ulrich (46); Gullone (48); Depledge et al. (49); Joye and van den Berg (50); Orr (51); and Tuan (52)
Social economics	Economic–nature conflicts	The values of nature are counter intuitive with those values and actions of capitalism. Examples used in this review related to natural resource management Overlaps identified between the following research disciplines and fields: social economics, ecosystem accounting (see Impacts of the Human–Nature Relationship on Health), power relationships, conservation and resource management, affiliation to nature, and biophysical systems	Relph (55); Hay (56); Glacken (57); Buckeridge (60); Small and Jollands (61); Hardin (62); Van Vugt (65); and Ostrom (66)
Environmentalism	Power relationships	Those power relationships exerted by both nature and humanity. Examples used in this review related to conservation behaviors and management of the natural environment Overlaps identified between the following research disciplines and fields: economic–nature conflicts, conservation management, social and cultural behaviors, social health (see Social Health), affiliation to nature, and biophysical systems	Radkau (69); Richards (71); Whited (77); Hodder and Bullock (80); Tidball (81); and Adger et al. (82)

Our understanding of the human–nature relationship and its underlying mechanisms could be further understood from an interdisciplinary perspective. In essence, the human–nature relationship can be understood through the Biophilia concept of humanity’s affiliation with nature as well as related concepts and measures to describe human connectedness to nature (49–53). Equally, Orr’s (51) perspective that at the commonest level humans can acquire other affinities to or learn to love different elements than those of the natural world (e.g., technology and urban environments) adds to this understanding. Further, while humanity, and indeed nature also, has not entirely escaped change, it cannot be assumed that all have been shaped by evolutionary mechanisms (42, 44). Some have been shaped by what Radkau (75) terms as the power shift between humans and nature, which is evolving, as it has and will keep on doing. As such, the human–nature relationship goes beyond the extent to which an individual believes or feels they are part of nature. It can also be understood as, and inclusive of, our adaptive synergy with nature as well as our longstanding actions and experiences that connect us to nature. Over time, as research and scientific knowledge progresses, it is anticipated that this definition of the human–nature relationship will adapt, featuring the addition of other emerging research fields and avenues.

## Defining Health

Conceptualizing “health” has often generated complex debates across different disciplines owing to its multidimensional and dynamic nature (83). It is, however, beyond the scope of this paper to review the many ways these concepts have been previously explored (84–86). Instead, “health” is reviewed and viewed more generally through the lens of the World Health Organization 1948 definition.

The World Health Organization defined “health” simply as the physical, social, and mental well-being of humanity, in which “health” was widened beyond those biomedical aspects (e.g., disease and illness) to encompass the socioeconomic and psychological domains (85). This classical definition advocated health’s shift toward a holistic perspective, with emphasis on more positive attributes (84, 87) and was not simply “*the mere absence of disease and infirmity*” [(83), p. 1]. It also reflected people’s ambitious outlook after the Second World War, when health and peace were seen as inseparable (83, 84). Since then, this shift has seen a major growth in the last 30 years, primarily in areas of positive health and psychology (88–92).

Despite its broad perspective of human health, the definition has also encountered criticism in relation to its description and its overall reflectance of modern society. For instance, the use of the term “completeness” when describing optimal health has been regarded by many as impractical. Instead, Huber et al (83) propose health to be the “ability to adapt and to self-manage” and invite the continuation of further discussions and proposals of this definition to be characterized as well as measured through its three interrelated dimensions; physical, mental, and social health. Similarly, others have highlighted the need to distinguish health from happiness (84) or its inability to fully reflect modern transformations in knowledge and development (e.g., technology, medicine, genomics as well as physical and social environments) (86). As such, there have been calls to reconceptualize this definition, to ensure further clarity and relevance for our adaptive societies (83).

Broadly, health has been measured through two theoretical approaches; subjective and objective (85). The subjective approach is based on individual’s perceived physical, emotional, and cognitive experiences or functioning. By contrast, the objective approach measures those variables, which are existing and measurable external to an individual’s internal experience such as living conditions or human needs that enable people to lead a good life (e.g., health markers, education, environment, occupational attainment, and civic involvement) (85). Together, these approaches provide a more comprehensive picture of a person’s health status, which are applicable across its three health components (physical, mental, and social), as described below.

First, physical health is defined as a healthy organism capable of maintaining physiological fitness through protective or adaptive responses during changing circumstances (83). While it centers on health-related behaviors and fitness (including lifestyle and dietary choices), physiological fitness is considered one of the most important health markers thought to be an integral measure of most bodily functions involved in the performance of daily physical exercise (93). These can be measured through various means, with examples including questionnaires, behavioral observations, motion sensors, and physiological markers (e.g., heart rate) (94).

Second, mental health is often regarded as a broad concept to define, encapsulating both mental illness and well-being. It can be characterized as the positive state of well-being and the capacity of a person to cope with life stresses as well as contribute to community engagement activities (83, 95). It has the ability to both determine as well as be determined by a host of multifaceted health and social factors being inextricably linked to overall health, inclusive of diet, exercise, and environmental conditions. As a result, there are no single definitive indicators used to capture its overall measurement. This owes in part to the breadth of methods and tends to represent hedonic (e.g., life satisfaction and happiness) and eudaimonic (e.g., virtuous activity) aspects of well-being, each known to be useful predictors of physical health components (96).

Third, social health can be generalized as the ability to lead life with some degree of independence and participate in social activities (83). Indicators of the concept revolve around social relationships, social cohesion, and participation in community activities. Further, such mechanisms are closely linked to improving physical and mental well-being as well as forming constructs, which underline social capital. Owing to its complexity, its measurement focuses on strengths of primary networks or relationships (e.g., family, friends, neighborliness, and volunteering in the community) at local, neighborhood, and national levels (97).

## Current Knowledge on the Human–Nature Relationship and Health

This section summarizes existing theoretical and literature research at the intersection of the human–nature relationship and health, as defined in this review. This has been explored through three Subsections “Physical Health,” “Mental Health,” and “Social Health.” It aims to identify areas of convergence as well as gaps and limitations.

### Physical Health

Though it is widely established that healthy eating and regular exercise have major impacts on physical health (98), within the past 30 years research has also identified that exposure to nature (e.g., visual, multisensory, or by active engagement) is equally effective for regulating our diurnal body rhythms to ensure physical vitality (99). Such notion stems from Wilson’s (44) proposed “Three Pillars of Biophilia” experience categories (Nature of Space, Natural Analogs, and Nature in Space), which relate to natural materials and patterns experienced in nature, inducing a positive impact on health (9). Empirical research in this domain was first carried out by Ulrich (46) who found that those hospital patients exposed to natural scenery from a window view experienced decreased levels of pain and shorter recovery time after surgery. Following this, research in this academic field has grown exponentially and encompasses a large literature base on nature’s health benefits. These include improvements in neurological and circadian rhythms relating to exposures to natural sunlight (100, 101), undergoing “Earthing” or physical contact with the Earth’s surface regulates diurnal body rhythms (102) as well as walking activities in forest environments reducing blood pressure levels (8).

In spite of its increasing findings, some have suggested the need for further objective research at the intersect of nature-based parameters and human health (9). One reason for this is that most studies have yet to be scrutinized to empirical scientific analysis (55, 103) owing to the research area’s reliance on self-reported measures with the need for inclusion of more quantitative forms of data (e.g., physiological and biochemical indicators). This presents inherent difficulty in comparing assessment measures or different data types relative to the size and scale of the variables being evaluated (9). Further, there still remain evidence gaps in data on what activities might increase levels of physical health as well as limited amount of longitudinal datasets from which the frequency, duration, and causal directions could be inferred (104).

### Mental Health

Mental health studies in the context of connecting with nature have also generated a growing research base since the emergence of the Biophilia concept in the mid-1980s (45). Much of its research within the Evolutionary Psychology discipline examines the recuperative effects of nature on well-being and its beneficial properties following researcher’s arguments of humanity’s affiliation for nature (105). Supporting research has been well documented in literature during the last few decades. These include “Heraclitean motion” or natural movement (14), natural sounds (106), children’s engagement activities within green settings (7, 107) as well as esthetic preferences for nature and natural forms (4, 49).

Criticisms of this research area center on the inability to decipher causal effects and direction of such benefits and in part relates to its predominant focus on “recuperative measure” than that of detecting its “source” (105). In light of this, reviewers repeatedly remark on researchers’ tendencies to focus on outcomes of well-being, neglecting the intervening mechanisms that sustain or inhibit well-being (108). Similarly, further mixed-method approaches and larger sample sizes are needed in this research field. This would enhance existing evidence gaps to enhance existing knowledge of variable interlinkages with other important sources (e.g., physical and social health aspects) as well as the diversity that exists between individuals (104).

### Social Health

In the last two decades, the relationship between people and place in the context of green spaces has received much attention in academic literature in regards to its importance for the vitality of communities and their surrounding environments (109). As studies have shown, the presence of green space can promote social cohesion and group-based activities, aspects that are crucial for maintaining social ties, developing communities, and increasing individual’s well-being (e.g., horticulture and ecological restoration) (110). Examples of findings include usage of outdoor space exponentially increases with number and locality of trees (111), children’s activities in green spaces improves social development (7) as well as accessibility to green spaces enhances social bonds in communities (112).

One of the main limitations within this field relates to the generally perceived idea that public green spaces are freely open to everyone in all capacities (113). This limitation has been, as already, highlighted from the emerging arguments in the field of environmental justice and economic–nature conflicts (63). As such, many researchers highlight the need to maintain awareness of other barriers that might hinder cohesion and community participation (e.g., semi-public space and social exclusion). Further, there still remains a gap between academic research and local knowledge, which would otherwise lead to more effective interventions. However, without implementing participatory engagement, many studies risk misrepresenting the true social, economic, and political diversity that would increase both our understanding of “real life” problems of concern as well as bringing depth to data collected (114). Nonetheless, for such approach to be implemented requires sufficient time, cost, and an adequate scale of resources to ensure for aspects of coordination, communication, and data validation (115).

## Impacts of the Human–Nature Relationship on Health

During the past four decades, researchers, health practitioners, and environmentalists alike have begun to explore the potential link between the human–nature relationship and its impact people’s health (10). This in part owes to the increasing evidence accumulating in research literature centering on the relationships between the following areas: chronic diseases and urbanization, nature connectedness and happiness, health implications of contemporary society’s lifestyle choices as well as the adverse impacts of environmental quality on the health of humans and non-humans alike (116, 117).

Such health-related effects that have been alluded to include chronic diseases, social isolation, emotional well-being as well as other psychiatric disorders (e.g., attention deficit disorders and anxiety) and associated physical symptoms (7, 118). Reasons for these proposed links have been suggested to stem from various behavioral patterns (e.g., unhealthy diets and indoor lifestyles) associated with consumerism, urbanization, and anthropogenic polluting activities (117, 119). Further, these suggested links have been inferred, by some, to be visible in other species (e.g., insects, mice, and amphibians) as a consequence to living in unnatural habitats or enclosures (120–122). Nonetheless, research within this field remains speculative with few counter examples (e.g., some species of wildlife adapting to urban environments), requiring further empirical analysis (108).

With a growing trend in the number of chronic diseases and psychiatric disorders, costs to the U. K.’s National Health Service (NHS) could rise as the use of prescriptive drugs and medical interventions increases (123). However, this anticipated trend is considered to be both undesirable and expensive to the already overwhelmed health-care system (124). In concurrence are the associated impacts on health equity (125, 126), equating to further productivity and tax losses every year in addition to a growing gap in health inequalities (127).

Furthermore, population growth in urbanized areas is expected to impact future accessibility to and overall loss of natural spaces. Not only would this have a direct detrimental effect on the health of both humans and non-humans but equally the functioning and integrity of ecosystem services that sustain our economic productivity (128). Thereby, costs of sustaining our human-engineered components of social–ecological systems could rise, having an indirect impact on our economic growth and associated pathways connecting to health (129, 130). As such, researchers have highlighted the importance of implementing all characteristics when accounting ecosystem services, particularly the inclusion of natural and health-related capital, as well as their intervening mechanisms. This is an area, which at present remains difficult to synthesize owing to fragmented studies from a host of disciplines that are more conceptually rather than empirically based (131).

## Toward an Interdisciplinary Perspective of Human and Ecosystem Health

Since the late nineteenth century, a number of descriptive models have been developed to encapsulate the dimensions of human health and the natural environment as well as their interrelationships (17). These include the Environment of Health (11), the Mandala of Health (12), the Wheel of Fundamental Human Needs (13), and the Healthy Communities (14). As VanLeeuwen et al (17) highlight in their review, each have not fully incorporated all relevant characteristics of ecosystems (e.g., multiple species, trade-offs, and feedback loops, as well as the complex interrelationships between socioeconomic and biophysical environments). Further, the Bioecological systems theory model encapsulates the biopsychological characteristics of an evolving theoretical system for scientific study of human development over time (16, 132). However, the model has been suggested by some (133, 134) to be static and compartmentalized in nature, emphasizing instead the importance of evolving synergies between biology, culture, and technology.

More recently, the concept “One Health” has gradually evolved and increased with momentum across various disciplines (15). It is broadly defined as the attainment of optimal health across the human–animal–environmental interfaces at local, national, and global levels. It calls for a holistic and universal approach to researching health, an ideology said to be traceable to pathologist Rudolf Virchow in 1858 (18). Yet, the concept has received criticisms regarding its prominence toward the more biological phenomena (e.g., infectious diseases) than those of a social science and spatial perspective (18, 135). Some have therefore suggested its need to adopt an interdisciplinary approach to facilitate a deeper understanding of the complexities involved (13).

**Figure 1:**
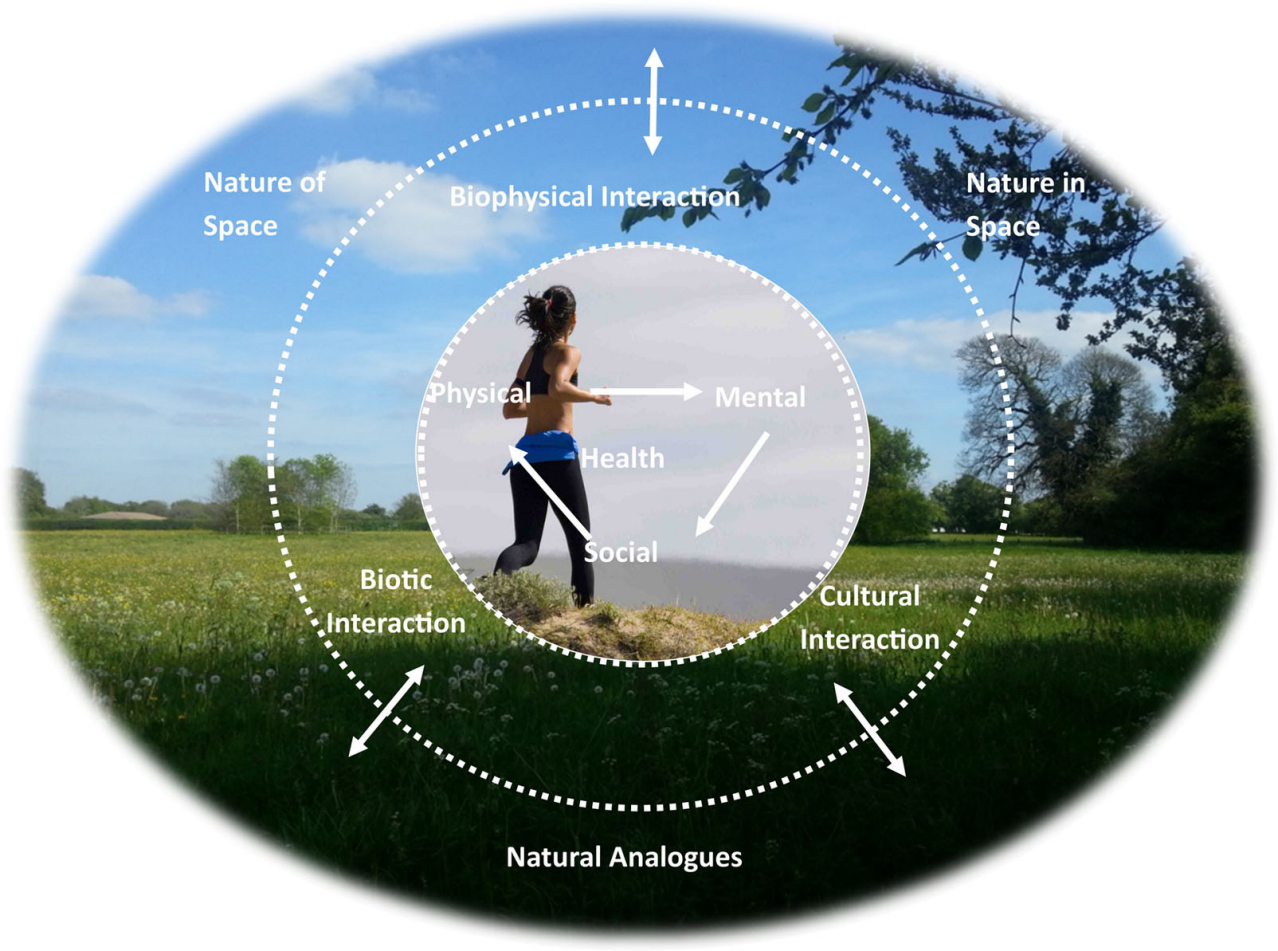
**Interdisciplinary perspective of human and ecosystem health [image on the inside circle is by Baird (136) with the background image, added text, and embedded illustrations being the author’s own work]**.

To address these limitations identified in the above models, a suggested conceptual model has been outlined below (Figure 1). It is both inclusive of all relevant characteristics of ecosystems, their continuously evolving synergies with human health as well as a balance between the biological, social, and spatial perspectives. This is achieved through combining the perspective of the human–nature relationship, as summarized in Section “Defining the Human–Nature Relationship” of this review, with those human-centered components of health (physical, mental, and social), as defined by the World Health Organization in 1948 in Section “Defining Health.” It aims to facilitate a deeper understanding of the complexities involved for attaining optimal human health (19). I will now describe the conceptual model.

First, the outer circle is representative of “nature” that both encompasses and interconnects with the three human-centered components of health (physical, mental, and social). Through this it emphasizes humanity’s interrelationship with the environment. As identified in Section “Defining the Human–Nature Relationship” of this review, the human–nature relationship can be experienced through various biological, ecological, and behavioral connections. For instance, social, political, and economic issues stemming from humanity’s interactions affecting the natural environment (e.g., natural resources, environmental hazards, habitat management, and restoration), as explored in Subsections “Social Economics” and “Environmentalism.”

Second, in the inner circle, the three components of human health (physical, mental, and social) are interconnected through a cohesive triangle to reflect their interdisciplinary and dynamic natures, as outlined in Section “Defining Health.” Further, this cohesive triangle acts on two levels. First, as a single construct of health based on these components combined. Second, the underlying intervening mechanisms that sustain or inhibit health, which can derive from each of these separately (105). Thereby, it not only focuses on the outcomes or “recuperative measure” of health but also the source of such outcomes and their directions, as highlighted in Section “Mental Health” (104).

The middle circle represents the interconnected relationship between humanity and the natural environment with relevance to human health (see Current Knowledge on the Human–Nature Relationship and Health). This has been indicated by the two-way arrows and incorporates Gual and Norgaard’s (31) coevolutionary perspective between human adaptation and the natural environment. In this way, the relationship is continually interconnected *via* two-way physical and perceptual interactions. These are embedded within three integrated systems (biophysical, biotic, and cultural), with all humanity knows of the world comes through such mediums (31). As such, the human–nature relationship goes beyond the extent to which an individual believes or feels they are affiliated with nature (e.g., Biophilia concept). It can also be understood as, and inclusive of, our adaptive synergy with nature as well as our longstanding actions and experiences that connect us to nature.

Utilizing this developing conceptual model, methodological approaches can be employed from those research fields explored in this review, enabling a more interdisciplinary framework. The characteristics, descriptions, implications, and practicalities of this are detailed in Table 2 below. The advantage of this is that a multitude of knowledge from both rigorous scientific analysis as well as collaborative participatory research can be combined bringing a greater depth to data collected (114). This could be achieved through using more mixed-method approaches and adopting a pragmatic outlook in research. In this way, the true social, economic, and political diversity of “real life” as well as the optimal human health at the human–environmental interface can be identified. As such, a more multidimensional perspective of human health would be gained, knowledge that could be implemented to address those issues identified in Section “Impacts of the Human–Nature Relationship on Health” (e.g., improving nature and health ecosystem service accounting). Nonetheless, adopting a pragmatic outlook brings its own challenges, as explored by Onwuegbuzie and Leech (137), with several researchers proposing frameworks that could be implemented to address these concerns (138, 139).

**Table 2:** **A summarized overview of human and ecosystem health from an interdisciplinary perspective**.

	Characteristics	Description	Implications and practicalities
Human health (inner circle)	Physical, mental, and social health	The three components of human health (see Defining Health): physical, mental, and social	This acts on 2 levels: collectively and intervening mechanisms
			To identify and evaluate the sources, directions as well as outcomes of health. To measure these through both objective and subjective indicators, using a mixed-method approach. Examples include questionnaires, governmental and public datasets, behavioral observations, and physiological markers
			To enhance understanding and accounting of health capital as well as intervening mechanisms. To use such knowledge to foster and support healthy lifestyles and communities

Human–nature relationship (middle circle)	Biophysical, biotic, and cultural interaction	Describes humans’ connections with the natural environment (see Defining the Human–Nature Relationship) and the interrelationship between two or more inherent systems (e.g., biophysical, biotic, and cultural)	This refers to a two-way relationship between human health and nature
		These connections were explored and summarized from those four research fields, which have paid most attention to studying the interface of humanity and the natural environment: evolutionary biology, evolutionary psychology, social economics, and environmentalism	To identify and evaluate the sources, directions as well as outcomes of these 4 human–nature connections, using an interdisciplinary perspective. To measure these through both objective and subjective indicators, using a mixed-method approach. Examples include participatory research methods, governmental and public datasets, as well as systematic and thematic reviews
			To enhance ecosystem services accounting, to be inclusive of natural and health-related capital. To integrate nature-based activities into health-care systems. To design human environments, social economic systems, and “power” relationships to be more in balance with nature

Nature (outer circle)	Nature in space, nature of space, and natural analogs	Describes humanity’s exposure to nature and experience categories, which relate to natural materials and patterns experienced in nature, both visually and non-visually (see Current Knowledge on the Human–Nature Relationship and Health and Impacts of the Human–Nature Relationship on Health)	Exposure refers to those visual, multisensory, or by active engagement
			To identify and evaluate the sources, directions as well as outcomes of exposure to nature. To measure these through both objective and subjective indicators, using a mixed-method approach. Examples include interviews, governmental and public datasets, and questionnaires
			To enhance understanding and accounting of natural capital as well as intervening mechanisms. To include such knowledge in human practices (e.g., public policies) and design

## Summary and Conclusion

One of the imperatives for this article is to review existing theoretical and research literature on the many ways that humans are linked with the natural environment within various disciplines. Although widely discussed across the main four research fields – evolutionary psychology, environmentalism, evolutionary biology, and social economics – there has been comparatively little discussion of convergence between them on defining the human–nature relationship. This paper therefore attempts to redefine the human–nature relationship to bring further understanding of humanity’s relationship with the natural environment from an interdisciplinary perspective. The paper also highlights important complex debates both within and across these disciplines.

The central discussion was to explore the interrelationships between the human–nature relationship and its impact on human health. In questioning the causal relationship, this paper addresses existing research on potential adverse and beneficial impacts in relation to humanity’s degree of relationship to nature and lifestyle choices. The paper also acknowledged current gaps and limitations of this link relative to the different types of health (physical, mental, and social), as characterized by the World Health Organization in 1948. Most of these relate to research at the intersect of nature-based parameters and human health being in its relative infancy. It has also been highlighted that the reorientation of health toward a well-being perspective brings its own challenges to the already complex research base in relation to its concept, measurement, and strategic framework. For a deeper sense of understanding and causal directions to be identified requires further attention to the complexities of these aspects’ interlinkages, processes, and relations.

Finally, a developing conceptual model of human and ecosystem health that is inclusive of the human-centered perspective is proposed. It is based on an interdisciplinary outlook at the intersection of the human–nature relationship and human health, addressing the limitations identified in existing models. To achieve this, it combines theoretical concepts and methodological approaches from those research fields examined in this review, bringing a greater depth to data collected. In attempting this, a balance between both rigorous scientific analysis as well as collaborative participatory research will be required, adopting a pragmatic outlook. In this way, an interdisciplinary approach can facilitate a deeper understanding of the complexities involved for attaining optimal health at the human–environmental interface.

## Author Contributions

The author confirms being the sole contributor of this work and approved it for publication.

## Conflict of Interest Statement

The author declares that the research was conducted in the absence of any commercial or financial relationships that could be construed as a potential conflict of interest.
